# 典型肺纤毛黏液结节性乳头状肿瘤1例临床病理分析

**DOI:** 10.3779/j.issn.1009-3419.2019.11.08

**Published:** 2019-11-20

**Authors:** 勇 张, 世春 陆, 霄霖 王, 露 樊, 兰巍 欧阳, 余声 束

**Affiliations:** 1 116000 大连，大连医科大学 Dalian Medical University, Dalian 116000, China; 2 225001 扬州，苏北人民医院胸外科 Department of Thoracic Surgery, Northern Jiangsu People' s Hospital, Yangzhou 225001, China

**Keywords:** 肺肿瘤, 肺纤毛黏液结节性乳头状肿瘤, 临床病理学, 免疫表型, Lung neoplasms, Ciliated muconodular papillary tumor of the lung, Clinicopathology, Immunophenotype

## Abstract

**背景与目的:**

肺纤毛黏液结节性乳头状肿瘤（ciliated muconodular papillary tumor of the lung, CMPT）的发病极其少见，在临床上与其他肺部病变亦难以区别，易造成误诊、漏诊；通过收集CMPT的资料，分析其临床病理特征，可以为广大医务工作者提供诊治思路，减少医疗差错。

**方法:**

回顾性分析1例典型的CMPT患者的临床资料、病理特征、免疫表型并结合相关文献进行探讨。

**结果:**

患者胸部计算机断层扫描（computed tomography, CT）提示右下肺近胸膜处可见混合密度结节影，直径约9 mm，肿瘤行肺楔形切除术，镜下见结节由增生的纤毛细胞、黏液细胞及基底样细胞混合组成，以乳头状、腺样结构为主，纤毛细胞衬覆于乳头状结构表面，基底样细胞位于外层，黏液细胞则位于两者之间，各种细胞异型不明显。免疫组化：上皮细胞CEA（+）、CK7（+）、CA125（+）、TTF-1（弱+）、CK20（-）、Ki67（1%+）、CK5/6（+）；基底细胞P63（+）。

**结论:**

CMPT是一种新近发现的罕见的肺部肿瘤，关于其良恶性目前尚无定论，但多数学者倾向于良性，其在影像学上可表现出诸多恶性肿瘤征象而常被误认为是肺腺癌，通过其典型的病理组织学特点及免疫组化表型可与其他肺部疾病进行鉴别，基因突变是否是其驱动因素目前尚不得知，该肿瘤行手术切除预后较好。

## Abstract (en)

**Background and objective:**

The pathogenesis of a ciliated muconodular papillary tumor (CMPT) of the lung is extremely rare which is difficult to distinguish from other lung lesions and it is easy to cause misdiagnosis and missed diagnosis. By collecting CMPT data, its clinical and pathological features can provide medical treatment ideas for the majority of medical workers and reduce medical errors.

**Methods:**

The clinical data, pathological features, immunophenotype of a typical CMPT patient and related literature were analyzed.

**Results:**

The chest computed tomography (CT) showed there was a mixed density nodule in the right lower lung near the pleura with a diameter of about 9 mm. We performed a wedge resection on the patient. The pathological results showed that the nodule was composed of proliferated ciliated cells, mucous cells, and basal-like cells. The ciliated cells were lined on the surface of papillary structures. The basal-like cells were located in the outer layer, while the mucous cells were located between the two. The cell atypia was not obvious. Immunohistochemistry: epithelial cells CEA (+), CK7 (+), CA125 (+), weakly positive for TTF-1, CK20 (-), Ki67 (1%+), CK5/6 (+), and basal cells P63 (+).

**Conclusion:**

CMPT is a rare pulmonary neoplasm. There is no definite conclusion about its biologic nature, but most experts prefer a benign to a malignant tumor. CMPT can show many malignant tumor signs on imaging and is often mistaken for lung adenocarcinoma. According to its typical histopathological characteristics and immunohistochemical phenotype, it can be differentiated from other pulmonary diseases. Whether gene mutation is the driving factor is still unknown. Surgical resection for the tumor reveals a good prognosis.

肺纤毛黏液结节性乳头状肿瘤（ciliated muconodular papillary tumor, CMPT）是一种罕见的肺部肿瘤。临床工作中，病理科医师和胸外科医师对其缺乏充分的认识，易将其误诊为腺癌而导致过度治疗。本文通过对我院首例CMPT患者进行回顾性分析，并结合相关文献对该肿瘤的诊断治疗进行深入探讨，以提高对该肿瘤的认识，避免临床工作中出现误诊。

## 1 材料与方法

### 1.1 临床资料

男，55岁，因“体检发现肺结节8个月”入院，患者无胸闷胸痛、无咳嗽咳痰、无畏寒发热等症状，既往体健，无肿瘤家族史及个人吸烟史，体格检查及相关实验室检查未见异常，8个月间随访两次胸部计算机断层扫描（computed tomography, CT），结节无明显变化，本次入院查CT报告：右下肺近胸膜处可见混合结节影，密度不均，大小约9 mm×9 mm，边界清晰，结节内存在不规则厚壁空洞，周围胸膜稍增厚（图 1A），入院完善相关检查后行右下肺楔形切除术。术后患者恢复良好，手术5 d后出院。

**1:**
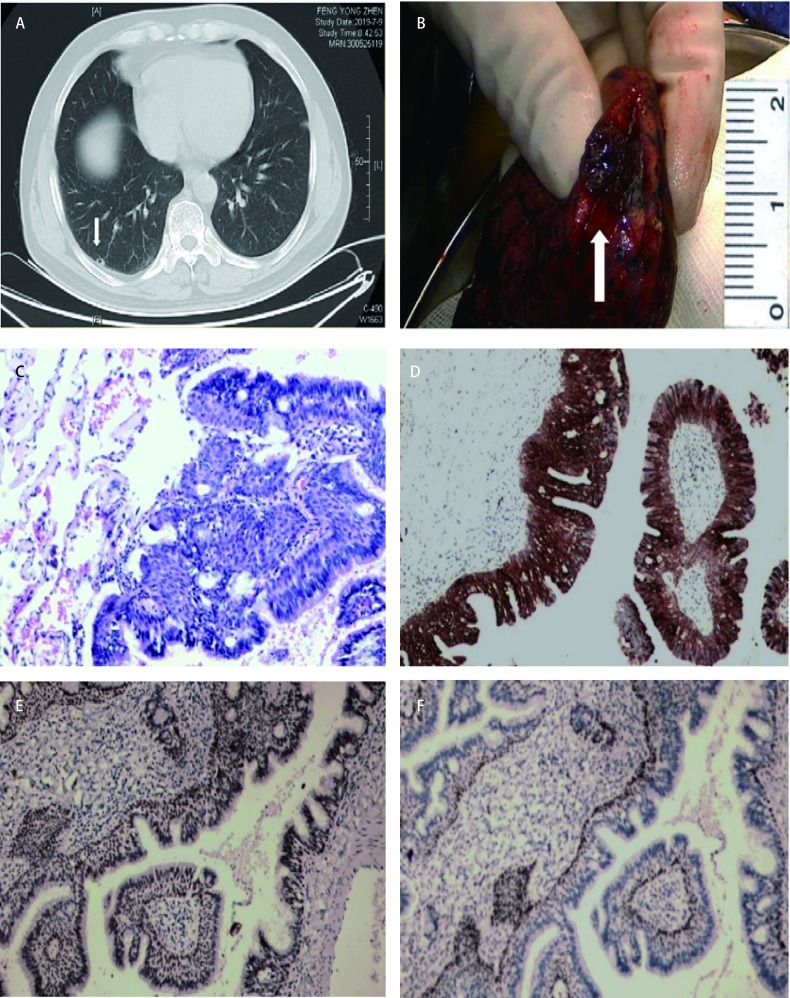
病例图片。A：CT影像上可见右下肺近胸膜处可见边界清晰的混合结节影，密度不均，大小约9 mm×9 mm，内可见不规则厚壁空洞，周围胸膜稍增厚（箭头处）；B：切开肿瘤见切面灰褐、质中、境界较清楚，具有黏液，大小约12 mm×5 mm×4 mm（箭头处）；C：镜下观见结节由增生的纤毛细胞黏液细胞及基底样细胞混合组成(HE染色法，×100)；D：纤毛细胞、黏液细胞和基底细胞CK7（+）(EnVision法，×100）；E：纤毛细胞、黏液细胞和基底细胞TTF-1（弱+）（EnVision法，×100）；F：基底细胞P63（+）（EnVision法，×100）。 Case pictures. A: CT images showed visible mixed nodules with clear borders in the right lower lung near the pleura. The density was uneven, and the size was about 9 mm×9 mm. Irregular thick-walled cavities were visible inside, slightly thickened around the pleura (arrow); B: Cut the tumor and see the cut surface gray brown, medium and clear, with clear mucus, and the size was about 12 mm×5 mm×4 mm (arrow); C: Microscopically, the nodules are composed of a mixture of proliferating ciliated mucous cells and basal cells (HE staining, ×100); D: Ciliated cells, mucous cells and basal cells CK7 (+) (EnVision method, ×100); E: TTF-1 weakly positive for ciliated cells, mucous cells, and basal cells (EnVision method, ×100); F: Basal cell P63 (+) (EnVision method, ×100). CT: computed tomography.

### 1.2 方法

标本取出后，经4%中性甲醛固定，常规取材、脱水后石蜡包埋，4 μm厚切片，行HE染色。免疫组化采用EnVision法，采用即用型抗体包括CEA、TTF-l、CK5/6、p63、CK7、CK20、和Ki-67（Dako）Napsin A。

## 2 结果

### 2.1 外观

在楔形切除的肺组织紧接胸膜处，距切缘约3 cm见一结节，大小12 mm×5 mm×4 mm，切面灰褐、质中、境界较清楚，具有黏液（图 1B）。

### 2.2 镜检

结节由增生的纤毛细胞、黏液细胞及基底样细胞混合组成，以乳头状、腺样结构为主，纤毛细胞衬覆于乳头状结构表面，基底样细胞位于外层，黏液细胞则位于两者之间，各种细胞异型不明显（图 1C）。

### 2.3 免疫组化

上皮细胞CEA（+）、CK7（+）（图 1D）；CA125（+）、TTF-1（弱+）（图 1E）；CK20（-）、Ki67（约1%+）、CK5/6（+）；基底细胞P63（+）（图 1F）。

## 3 讨论

根据其组织形态学及临床表现，有研究^[1]^于2002年对该病例进行了首次报道并将其命名为CMPT，截止目前，国内外相关个案报道仅约数十例，是一种较为罕见的肺部肿瘤。2015年世界卫生组织（World Health Organization, WHO）呼吸系统肿瘤分类中未对该肿瘤进行描述；该病主要发生在东方人群，西方人中亦可见少量发病^[2]^，男性相对多见，而是否吸烟与该病并无明显相关性，患者中又以中老年人较为多见，但亦有青少年罹患该病的报道^[3]^，该病在临床上一般无特殊症状，多由体检或其他疾病进行检查时偶然发现。

### 3.1 诊断

CMPT在外观上通常为灰白或灰褐色、柔软、境界清楚的结节，具有黏液或凝胶质^[4]^，影像学上常表现为单发的、位于肺外周的小结节，肿瘤直径通常≤15 mm^[5]^，可见毛刺、胸膜凹陷等征象，中心可伴有不规则空洞，肿瘤界限相对清楚，形态规则或不规则^[6]^，因该肿瘤在影像学上具有多种恶性征象，故CT检查时其常被误诊为肺腺癌。

镜下可见到该肿瘤具有特征性的生长模式，如腺泡、乳头状、微乳头状，甚至肿瘤沿着肺泡壁进行增殖，周围肺泡腔内充满黏液；肿瘤由纤毛柱状细胞、黏液细胞和基底细胞所组成，纤毛柱状细胞位于腺样及乳头样结构的表面，基底细胞位于外层起支架作用，而黏液细胞则散在分布；镜下肿瘤细胞没有见到明显异型性、核分裂象（有丝分裂）及坏死，肿瘤周围或内部可见大量肺泡腔内黏液，部分肿瘤上皮沿肺泡壁呈不连续生长，周围无包膜，且多数病例伴有形成不规则的支气管壁或厚壁血管^[5]^，肿瘤间质有增生的纤维组织，常伴有淋巴细胞、浆细胞浸润。

免疫组化：CMPT的3种细胞表达CEA和CK7多强阳性，TTF-1弱阳性，Ki-67低表达（≤10%），CK20和MUC2阴性，基底细胞p40、p63和CK5/6强阳性，黏液细胞不表达MUC5AC，而纤毛细胞局部表达MUC5AC^[4, 7]^；本文报道的1例CMPT，肿瘤镜下病理特征及免疫组化与相关报道基本符合。

CMPT内存在多项基因突变，Kamata等^[8]^报道10例中有8例（80%）发生突变，其中4例具有*BRAF*-V600E基因突变，1例携带*BRAF*-G606R突变，3例检测出EGFR第19号外显子缺失；Liu等^[2]^认为*BRAF* V600E有致癌特性且其在广泛的人类肿瘤驱动中具有重要作用，其被认为是约40%的CMPT中的重要驱动突变；与此同时，Liu等^[2]^对报道的CMPT病例进行基因检测，其中1例显示有*BRAF*-V600E和*AKT1*-E17K突变；Udo等^[7]^报道的4例CMPT中，2例检测到基因突变，1例为*BRAF*-V600E和*AKT1*-E17K突变，另1例首次检测到*KRAS*-G12D突变。然而，鉴于CMPT的病例报道数较少，针对其基因突变的研究尚不足，所以目前关于该肿瘤的驱动基因尚不能完全明确，但以上报道足可证明CMPT是一种肿瘤性病变而非反应性的化生性改变。

### 3.2 鉴别诊断

鉴于CMPT的罕见性及其组织病理学、免疫组化方面的独特性，临床诊疗中需注意与以下几种病变相鉴别。

#### 3.2.1 黏液腺癌

部分CMPT细胞沿肺泡壁不连续生长，且形成腺管、乳头状结构，肿瘤内或周边又充满黏液，容易与黏液腺癌相混淆^[9]^；但CMPT镜下由典型的纤毛柱状细胞、黏液细胞以及连续的基底细胞构成，且其肿瘤细胞无异型性及核分裂活性；而黏液腺癌镜下多无纤毛成分，可见到不同程度的细胞异形、核分裂相及坏死，不存在连续的基底细胞群，且其免疫组化TTF-1多为阴性^[10]^。基因表达方面亦可作为两者鉴别点，Kamata等^[4]^报道，CMPT经常（5/10）表达*BRAF*突变，但在肺腺癌中却很少有这种突变（3%）；通过以上几点对两者进行鉴别，具有相当的价值。Udo等^[7]^研究认为：CMPT的病变具有异质性，某些CMPT可能是侵袭性黏液腺癌的某种癌前病变，但该观点尚需要进一步研究来证实。

#### 3.2.2 肺黏液表皮样癌

该肿瘤起源于支气管粘膜的腺体及其导管，是一种低度恶性的真性肿瘤，由黏液样细胞、表皮样细胞和中间细胞组成，与CMPT有一定相似性，但其影像表现多为中央型，通常具有多个病灶，肿瘤镜下缺乏纤毛细胞，且细胞具有异型性，亦可与CMPT进行鉴别^[11]^。

#### 3.2.3 细支气管鳞状上皮化生

该病变细胞成分与CMPT类似，但CMPT一般为单病灶，而细支气管鳞状上皮化生往往形成多个病灶，且无复杂结构；再者，该病变的免疫组化MUC5AC呈阳性表达^[12]^，而CMPT的黏液细胞多不表达MUC5AC^[13]^。

#### 3.2.4 乳头状瘤

2015年世界卫生组织（World Health Organization, WHO）将支气管乳头状瘤收录在良性肿瘤范围，并将其分为鳞状细胞乳头状瘤、柱状细胞乳头状瘤及混合性乳头状瘤3种^[1]^，后两种尤其是混合性乳头状瘤与CMPT的组织结构及细胞成分非常相似；乳头状瘤发生在大的支气管腔内，并呈息肉状突入支气管腔，患者有气道阻塞的典型表现，而CMPT则发生与肺外周组织，即支气管腔外，且多无临床症状，但也有肺外周型的乳头状瘤的病例报道^[14, 15]^；Aida等^[16]^描述了3例肺外周型孤立纤毛腺性乳头状瘤（solitary peripheral ciliated glandular papillomas, SPCGP），该病例在2015年被WHO引用为罕见的发生于肺外周的腺性乳头状瘤，通过对比，两种肿瘤具有很相似的临床病理学特点，SPCGP是发生于肺外周的孤立性小结节且部分病例位于细支气管内，在镜下可见到乳头状、管状结构，被覆不同比例的纤毛细胞和黏液细胞，其下可见连续的基底细胞，因此，国内外部分学者认为CMPT与SPCGP是同一类肿瘤^[4, 17]^；根据WHO定义：混合性乳头状瘤在支气管内形成，所以，该肿瘤和CMPT的生长机制可能经历了相同过程，区别仅在于它们的位置（中央或外周）以及两者与支气管腔的关联（支气管内或支气管外）^[1]^；也有报道^[13]^称，混合乳头状瘤的黏液细胞MUC5AC阳性，而CMPT的黏液细胞MUC5AC为阴性；因此，乳头状瘤与CMPT究竟是不同病变还是同一病变的不同谱系，目前还存在一定的争议。

#### 3.2.5 具有纤毛结构的乳头状腺癌

一般而言，具有纤毛结构被认为是良性的标志，但也有少量具有纤毛结构的乳头状腺癌的病例报道^[18, 19]^，但此种腺癌的纤毛被覆上皮过于单一，且肿瘤细胞存在异型及核分裂像，以及免疫组化中CMPT的基底细胞表达P63及P40，都可用于鉴别二者。

### 3.3 CMPT良恶性判断

多数学者倾向于将CMPT归为良性病变，理由如下：目前发现的CMPT患者，无论行肺叶切除、肺段切除或肺楔形切除，术后经过长时间随访，皆未发现复发病例，肿瘤发展遵循着良性临床过程^[4]^；其次，CMPT组织学上具有纤毛，而纤毛的存在可作为支持良性诊断的可靠发现^[20]^；CMPT在镜下无异型性、核分裂象、坏死以及Ki-67的低表达均支持其为良性病变^[21]^；Kamata等^[8]^的研究表明：CMPT的BRAF高表达，而该基因在肺癌患者中很少发生突变（3%），在亚洲患者中更为罕见（1.3%），研究中，有3例患者表达了带有dele746-t751/s752v突变的*EGFR*基因，这在肺腺癌中也很少见，两种基因在CMPT及肺癌中的突变的对比亦提示CMPT的良性倾向。但有部分学者认为CMPT亦具有潜在的恶性倾向，该观点认为CMPT无包膜，正常肺泡结构被破坏，肿瘤沿肺泡壁的增殖以及其类似于原位腺癌的跳跃病变^[23]^，肿瘤形成黏液湖、微乳头及间质纤维组织增生，CEA（+），都提示其恶性可能；且虽然发现纤毛细胞往往被认为是病变的良性过程，但亦可见到具有纤毛的肺黏液腺癌的报道^[18, 19, 22]^。因此，目前CMPT的良恶性判断尚未明确，但支持良性诊断的证据多于支持恶性诊断，因此，临床上可将CMPT作为良性肿瘤予以诊治。

### 3.4 CMPT的治疗

就目前而言，肺楔形切除可作为主要推荐，系统性淋巴结清扫或采样的意义并不大，但在临床实践中，由于该病罕见，临床和病理医生对该疾病缺乏了解，术中冰冻病理往往亦难以准确识别病变的性质，CMPT经常被误诊为肺腺癌，所以目前该疾病合理的诊断和治疗方式仍在探索中^[23]^。

## 4 总结

CMPT作为一种新近发现的肺部肿瘤，由于其发病罕见，国内外报道的病例数并不多。本文通过描述我院首例CMPT患者的临床资料，并查阅国内外相关文献报道，对该疾病的诊断、病理及免疫组化特点、可能的基因改变以及鉴别诊断进行了详细叙述。通过本文，胸外科医师及病理科医师可提高对该疾病的认识，尽可能避免在临床工作中出现误诊。
